# IL-25-induced activation of nasal fibroblast and its association with the remodeling of chronic rhinosinusitis with nasal polyposis

**DOI:** 10.1371/journal.pone.0181806

**Published:** 2017-08-03

**Authors:** Soo-Kyoung Park, Yong-De Jin, Yeong-Kyu Park, Sun-Hee Yeon, Jun Xu, Rui-Ning Han, Ki-Sang Rha, Yong-Min Kim

**Affiliations:** 1 Department of Otorhinolaryngology-Head and Neck Surgery, Research Institute for Medical Science, Chungnam National University School of Medicine, Daejeon, Korea; 2 Department of Otorhinolaryngology-Head and Neck Surgery, Yanbian University Hospital, Yanji, China; Tongji Hospital of Tongji Medical College of Huazhong University of Science and Technology, CHINA

## Abstract

**Background and objective:**

Interleukin (IL)-25 has been shown to play an important role in the pathogenesis of chronic rhinosinusitis with nasal polyps. Nasal polyps are associated with chronic inflammation of the mucous membranes in the paranasal sinuses and are involved in extracellular matrix (ECM) accumulation. The aim of this study is to evaluate the effects of IL-25 on myofibroblast differentiation, ECM production and the expression of matrix metalloproteinases in nasal polyp derived fibroblasts (NPDFs) and to determine the molecular mechanism underlying these processes.

**Materials and methods:**

A total of 40 patients were enrolled in this study for Immunofluorescence studies. Expression of IL17 receptor B was evaluated by real time reverse transcription polymerase chain reaction (PCR) in NPDFs. NPDFs were stimulated with IL-25 for 48 h in the presence or absence of mitogen-activated protein kinase (MAPK) and NF-κB inhibitors or small interfering RNAs (siRNA). The protein levels of fibrosis active mediators were examined using western blotting. Fibroblast migration was evaluated with a scratch assay. The total collagen amount was analyzed with the Sircol collagen assay.

**Results:**

IL-25 induced α-SMA, fibronectin, and MMP-1 and -13, which were dependent on IL-17RB. IL-25 also induced activation of NF-κB and mitogen-activated protein kinase (MAPKs). By using the specific inhibitor of ERK, p38, JNK and NF-κB (U, SB, SP and Bay), we found that IL-25-induced expressions of α-SMA, fibronectin, and MMPs was regulated by the signaling pathways of MAPKs and NF-κB. IL-25 also induces α-SMA, fibronectin, and MMPs expression through IL-17RB-dependent pathways in NPDFs. The increased migration ability induced by IL-25 was suppressed by the specific inhibitors of MAPKs and NF-κB.

**Conclusion:**

Our data indicate that IL-25 induced myofibroblast differentiation, fibronectin production, and MMP-1 and -13 expressions through the signaling pathways of MAPKs and NF-κB. in NPDFs and increased expression of IL-25 were also involved in the pathogenesis of nasal polyposis by affecting nasal fibroblasts in chronic rhinosinusitis with nasal polyps.

## Introduction

Chronic rhinosinusitis with nasal polyposis (CRSwNP) is a chronic inflammatory disease of the paranasal sinuses whose underlying etiology is multifactorial in nature [1]. Nasal polyposis is histologically characterized by persistent inflammation and irreversible structural changes that lead to remodeling in the sinonasal mucosa [2]. We have previously shown that nasal polyps are composed of various cell types, including epithelial cells, fibroblasts/vascular endothelial cells, eosinophils, CD4+ T cells, CD8+ T cells, B cells, macrophages, mast cells, and dendritic cells. We previously reported that epithelial cells and fibroblasts, are two of the major components of the nasal polyp derived cells [3] and fibroblasts confer mechanical strength by providing a supporting framework for the extracellular matrix (ECM). Interleukin (IL) - 25 is mainly produced from epithelial cells, and fibroblasts are a cellular source of ECM protein. [4]. The components of the ECM play essential roles in inflammatory reactions and can also be the sites of numerous structural changes from fibrosis to extreme edema of the lamina propria [5, 6]. Myofibroblasts that express alpha-smooth muscle actin (α-SMA) comprise an activated cell phenotype of fibroblasts with a high capacity for ECM protein secretion and play an important role in ECM remodeling of many pathologic conditions of the human airway, including asthma, chronic rhinosinusitis, and nasal polyps [7, 8].

A member of the IL-17 cytokine family and mainly produced by the epithelium, IL-25 noticeably promotes Th2 cell-mediated inflammatory responses and can promote the recruitment of eosinophils, innate lymphoid cells, and mast cells to the inflammation site [9, 10]. Epithelial-derived IL-25 can also induce the epithelium to produce more IL-25 and other potent innate cytokines, such as IL-33 and thymic stromal lymphopoietin, thus intensifying the allergic inflammation [11]. In addition to driving Th2 inflammation, IL-25 expression is known to be involved in airway remodeling by mediating pulmonary collagen deposition, neovascularisation, peribronchial smooth muscle hyperplasia and airway hyperreactivity following allergen exposure [12]. According to a recent study, IL-25 protein levels were significantly increased in NP tissue homogenates from patients with CRSwNPs, and further analysis has shown that IL-25 secreted from the sinonasal epithelia and infiltrating mast cells plays a crucial role in the pathogenesis of CRSwNPs in Asian patients [3, 13].

IL-25 binds a receptor complex composed of IL-17RB (also known as IL-25R), which partners with IL-17RA [14, 15]. IL-17rb-/- and IL-17ra-/- mice fail to respond to IL-25, and both knockout strains are refractory to pulmonary inflammation induced by intranasal application of IL-25 [15, 16]. Recent studies have demonstrated that Act1 and STAT5 in the epithelium and in T cells play critical roles in IL-25-dependent type 2 responses for allergic lung inflammation [17, 18]. Aside from signaling through Act1 or STAT5, IL-25 has also been shown to activate MAPKs such as P38 and JNK as well as NF-kB [19]. Although the role of IL-25 in inducing type 2 allergic inflammatory responses and the components of its signaling cascade are well recognized, the effects of IL-25 on fibroblast activation and the mechanism underlying NPDFs has not yet been determined. Because epithelial cells and fibroblasts are the main components of nasal polyps, and epithelial-derived IL-25 expression has been shown to be significantly higher in nasal polyp tissues than in control nasal tissues, an investigation into the effects of IL-25 on NPDFs would be highly relevant to this field. This study aims to evaluate the effects of IL-25 on fibroblast activation and differentiation, ECM production and MMP expression in NPDFs and to determine the underlying molecular mechanism of these processes.

## Materials and methods

### Reagent

Human recombinant IL-25 was purchased from R&D System (Minneapolis, MN) and was dissolved in sterile 4 mM HCl containing at least 0.1% bovine serum albumin. U0126 (a specific inhibitor of ERK), SB203580 (a specific inhibitor of p38) and SP600125 (a specific inhibitor of JNK inhibitor for JNK1, JNK2 and JNK3) were obtained from Calibiochem (Billerica, MA). STAT5 inhibitor was purchased from Merck Millipore (Nottingham, United Kingdom). Bay 11–7082 (NF-κB inhibitor) and BX-795 (IRF3 inhibitor) were purchased from Sigma (St. Louis, MO). All inhibitors were dissolved in dimethyl sulfoxide. Sircol collagen assay kits were acquired from Biocolor Ltd (Belfast, N. Ireland, UK).

### Patients and tissue preparation

A total of 40 patients were enrolled in this study for immunofluorescence studies. Forty patients who visited the Department of Otorhinolaryngology of Chungnam National University Hospital in Korea between January 2016 and November 2016 were included in the study. Of the 40 patients, 20 had CRSwNP, 10 had CRS without NP (CRSsNP), and 10 subjects underwent other rhinologic surgeries, such as dacriocystostomy, or endoscopic orbital decompression surgery were enrolled as control subjects. Uncinate tissues (UT) were obtained from the 10 patients with CRSwNP, 10 patients with CRSsNP, and 10 control subjects. Polyp tissues were obtained from the NP of patients with CRSwNP.

A sinus disease diagnosis was based on patient history, clinical examination, nasal endoscopy, and computed tomography of the paranasal sinuses, as detailed by the guidelines contained in ‘‘EPOS 2012: European position paper on rhinosinusitis and NPs 2012” [1]. Patients who used oral or nasal corticosteroids or other medications (e.g., antibiotics or antileukotrienes) for 4 weeks before sample collection; those with recent upper respiratory tract infections; and patients undergoing surgical revision were excluded from the study.

Details of the patients’ characteristics are shown in Table 1. The median age of the patients was in the CRSwNP group than those in the control and CRSsNP groups. The 3 groups did not differ significantly with respect to gender, comorbity of bronchial asthma, atopy or aspirin intolerance. NP tissues from 5 patients with CRSwNP were used for the NPDF culture. A written informed consent was obtained from each patient and control subject before enrollment into the study. The study was approved by the Institutional Review Board of the Chungnam National University Hospital.

**Table 1:** Patient characteristics.

Group	Controls	CRSsNP	CRSwNP	*p* Value
Total of subjects, *n*	10	10	20	
Gender, male, *n* (%)	8 (80)	7(70)	16(80)	0.885
Age, median (IQR), year	37.3 (17.1)	43.0 (14.2)	54.3 * (16.3)	0.02*
Bronchial asthma, *n* (%)	0 (0)	1 (10)	2 (10)	0.798
Atopy, *n* (%)	0 (0)	0 (0)	1 (5)	1
Aspirin intolerence, *n* (%)	0 (0)	0 (0)	0 (0)	0.487
Lund-Mackay CT score, median (IQR)	0 (0)	7.0 (5.3)	13.5 (5.3)	ns

*Statistically significant (P < 0.05)

IQR = interquartilerange; ns = not significant

### NPDF culture

NPDFs were isolated from the surgical tissues by cutting them into small pieces with a sanitized scissor, followed by collagenase (500 U/ml, Sigma) digestion. The cells were cultured in Dulbecco’s Modified Eagle Medium containing penicillin G (10,000 U/ml), streptomycin (10,000 μg/mL), amphotericin B (25 μg/mL) and 10% fetal bovine serum (FBS) (Invitrogen, Carlsbad, CA, USA).

The NPDFs were seeded in culture plates for 7 days, after which the unattached cells were eliminated. The purity of the obtained fibroblasts was confirmed by characteristic spindle and bipolar or multipolar morphology as well as flow cytometry. The fourth cell passage provided every cell used for this research. NPDF isolate from 5 patients were used for in vitro experiment; including fluorescence activated cell sorting (FACS) analysis, siRNA, cell migration assay, real-time polymerase chain reaction (RT-PCR) and western blotting.

### Immunofluorescence studies

The paraffin-embedded tissue samples were soaked first in xylene to remove the paraffin wax and then sequentially in solutions of 100%, 95%, and 70% ethanol for rehydration. Antigen retrieval was performed by heating the slides in a Decloaking Chamber (Biocare Medical, Concord, CA) to 120°C. A protein block [10% normal chicken serum (Vector, Burlingame, CA, USA) in phosphate-buffered saline (PBS) and 0.3% Triton X-100 (Biosesang, Seongnam, Republic of Korea) for 1 hour at room temperature] was then applied to the tissue to prevent non-specific protein binding. Endogenous peroxidase activity was blocked by incubating the sections in 1% hydrogen peroxide solution (Sigma-Aldrich, St. Louis, MO, USA) in PBS with 0.3% Triton X-100 for 30 minutes at room temperature. Mouse anti-Vimentin antibody (Invitrogen, Carlsbad, CA, USA), rabbit anti-alpha SMA (Abcam, Cambridge, MA, USA), and rabbit anti-IL-25 (Abcam, Cambridge, MA, USA), at a concentration of 10μg/mL, were used as the primary antibody which was incubated with the tissue at 4°C overnight. The sections were rinsed 3 times with PBS and then incubated for 2 hours at room temperature with Alexa Fluor@ 488-conjugated goat anti-mouse IgG and Alexa Fluor@ 594-conjugated goat anti-rabbit (Life Technologies, Carlsbad, CA, USA) as a secondary antibody. After rinsing with PBS, 4',6-diamidino-2-phenylindole (DAPI; Invitrogen, Carlsbad, CA, USA) was used at a concentration of 300 nM for nuclear counterstaining. After final rinsing with PBS, the samples were mounted using Fluoro-Gel with Tris Buffer (Electron Microscopy Science, Hatfield, PA, USA). The slides were subsequently observed on a fluorescence microscope (Olympus, Tokyo, Japan). Vimentin/alpha-SMA and IL-25/ alpha-SMA double-positive cells were counted per high-powered field (HPF, × 400) at 3 different randomly chosen sites in the tissue, and the mean values with range were calculated.

### FACS

The NPDFs were stained with Vimentin (eBioscience, Ireland, United Kingdom) and alpha-SMA (eBioscience, Ireland, United Kingdom). 7-AAD and 7-amino-actinomycin D (Sigma-Aldrich, St. ILouis, MO, USA) were used for staining the nonviable cells in our flow cytometric analysis. In brief, the NPDFs were washed in PBS buffer, and the staining dyes Vimentin (eBioscience, Ireland, United Kingdom) and 7-AAD (Sigma-Aldrich, St. ILouis, MO, USA) were then added and incubated in the dark for 20 minutes on ice. The NPDFs were washed in PBS and then fixed with 4% paraformaldehyde. Through this process, more than 4 × 105 cells were prepared and subjected to FACS analysis.

### Immunohistochemical assays

The tissue samples were prepared through fixation in 4% buffered formalin, followed by embedding in paraffin and sectioning of the obtained paraffin block at every 4 μm thickness. Immunohistochemistry was performed using a modified streptavidin- biotinylated peroxidase technique with mouse anti-human vimentin (Affinity Bioreagents; Golden, CO), rabbit anti-human alpha-SMA (Abcam, Cambridge, MA, USA) and mouse anti-human IL-25 (Abcam, Cambridge, MA, USA).

The slides were prepared manually with pre-treatment by boiling them in sodium citrate buffer (pH 10) for 60 minutes, at 90°C. Nonspecific protein staining was blocked by goat serum for 30 min at room temperature. The sections were incubated with primary antibody at 4°C overnight. The sections were rinsed with PBS and incubated with biotinylated secondary antibodies for 60 min. They were then washed and treated with 0.3% hydrogen peroxide in methanol for 30 min to inhibit the activity of any endogenous peroxide. The slides were washed, incubated with streptavidin-biotin-peroxidase complex for 30 min, and developed according to the manufacturer’s protocol. The immunoreaction was subsequently visualized with 3, 3’-diaminobenzidine (DAB). After a final rinsing with PBS, the samples were mounted using Fluoro-Gel with Tris Buffer (Electron Microscopy Science, Hatfield, PA, USA).

The slides were subsequently observed on a fluorescence microscope (Olympus, Tokyo, Japan). Vimentin, alpha-SMA and IL-25 positive cells were counted per high-powered field (HPF, × 400) using the tissues from the three different patients, and the mean values with range were calculated.

### Application of Short Interfering RNA (siRNA)

A duplex siRNA designed to target human IL-17RB (NCBI: NM_018725) was purchased from Bioneer (Daejeon, Republic of Korea). A nonsilencing siRNA duplex targeting was used as the control siRNA (random siRNA duplex). The NPDFs were seeded in six-well plates at a density of 5 × 10^5^ cells per well to obtain 70 to 80% confluence. Transfection of the siRNA was performed with Lipofectamine® RNAiMAX (Cat 13778075; Life Technologies, Carlsbad, CA, USA) in culture medium (without FBS and antibiotics) according to the manufacturer’s instructions. Forty-eight hours after transfection of siRNA (at a concentration of 100 pmol/μL), RT-PCR and western blot analysis were performed to examine the gene silencing effect.

### Cytotoxicity assay

Cell cytotoxicity and viability of the IL-25 protein were measured by MTT (3-[4,5-dimethylthiazol-2-yl]-2,5-diphenyl-tetrazolium bromide, Sigma-Aldrich, St Louis, MO, USA). The NPDFs were seeded at a density of 5×10^4^ cells in 96-well plates. After incubation at 37°C in the presence of 5% CO_2_ in a constant temperature and humidity incubator for 1day, the NPDFs were treated with different doses of IL-25. After 72 h, 10μl of the MTT solution (5 mg/mL) was added 4 h before the end of the incubation duration, and the reaction was terminated by the addition of 100 μL of dimethylsulfoxide (DMSO). Another MTT assay was performed to exclude the possibility of specific inhibitors namely U (a specific inhibitor of ERK), SB (a specific inhibitor of p-38), SP (a specific inhibitor of JNK) and Bay (a specific inhibitor of NF-κB)-induced cellular cytotoxicity of the NPDFs. The optical density (OD) or absorbance was read at 570 nm. The experiments were performed at least 3 times in triplicate.

### Cell migration assay

To study the effects of IL-25 and inhibitor treatment on cell migration, a monolayer wound healing assay was performed. The NPDFs were seeded at a density of 1x10^4^ cells/well in a 35mm culture-insert 4 well μ-Dish (ibidi, Germany). Upon reaching complete confluence, the inner well of the μ-Dish was removed, and the detached cells and debris were washed 3 times with PBS. The cells were incubated in a serum free DMEM medium with IL-25 and/or inhibitor of ERK, p38, JNK (U0126 10μm, SB203580 10μm, SP600125 10μm) and NF-κB (Bay 11–7082, 1μm). Photomicrographs were taken at time 0 (immediately following the scratch wound), 24 and 48 hours under a phase-contrast microscope. For statistical analysis, 3 randomized microscopic fields containing the scratch in the center of the fields were captured for each culture dish. The number of fibroblasts located in the scratches was counted and the averages of the count numbers of the 3 randomized microscopic fields for each culture dish were calculated.

### RT-PCR

mRNA expression of IL-17 receptor B (IL-17RB) was evaluated using RT-PCR. Total RNA was isolated from the NPDFs, CRSwNP (NP), control (UT) and HeLa cell (negative control) using a TRIzol™ reagent (Invitrogen, Carlsbad, CA, USA), according to the manufacturer’s instructions. For cDNA synthesis, 1 μg of total RNA was transcribed with AccuPower™ RT PreMix (Bioneer, Daejeon, Republic of Korea), according to the manufacturer’s instructions. PCR was performed for cDNA synthesis using a T100^TM^ Thermal Cycler (Bio-Rad Laboratories, Hercules, CA, USA). The mRNA expression was analyzed using a CFX Connect ^TM^ Real-Time PCR Detection System (Bio-Rad Laboratories, Hercules, CA, USA) with PowerUp ^TM^ SYBR ^TM^ Green Master Mix (Applied Biosystems, Carlsbad, CA, USA). PCR was performed using the following primers: human IL-25R (IL-17BR) (sense sequence: 5′-AACAGGCGTCCCTTTCCCTCTGGA-3′ and antisence sequence: 5′-TTCTTGATCCTTTCGTGCCTCCAC-3′); glyceraldehyde-3-phosphate dehydrogenase (GAPDH) (sense sequence: 5’-GTG GAT ATT GTT GCC ATC AAT GAC C-3’ and antisense sequence: 5’-GCC CCA GCC TTC TTC ATG GTG GT-3’).

All PCR assays were performed in triplicate. For each sample, the differences in threshold cycles between the target molecules and GAPDH (ΔCttarget gene, ΔCtreference gene) were determined; a calibrated delta Ct value (ΔΔCt, ΔCttarget gene -ΔCtreference gene) was calculated; and the relative quantitation (RQ) values were then calculated using the following equation: RQ = 2-ΔΔCt.

### Sircol collagen assay

The Sircol Collagen Assay kit (Biocolor Ltd, UK) is a quantitative dye-binding method designed to analyze acid soluble collagens extracted from mammalian tissues and collagens released into culture medium by mammalian cells during in vitro culture. The assay was performed according to the manufacturer’s recommendations. The optical density of sirius red was read against a blank at a wavelength of 540 nm (OD540), and the results were expressed as collagen concentration (mg/mL). In some experiments, the cells were pretreated with U0126 (a MEK/ERK signaling inhibitor) or SB431542 (a ALK5/Smad2/3 signaling inhibitor) or SP600125 (an MEK/JNK inhibitor) or Bay 11–7082 (a NF-κB inhibitor) for 2 hours, after which human recombinant IL-25 was added and co-incubated for 3 days.

### Western blot analysis

NPDFs were treated with IL-25 and inhibitors for 48 h. A total of 5×10^5^ cells were lysed in a RIFA buffer (Cell Signaling Technology, USA) that contained protease inhibitors (iNtRON Biotechnology, Korea) and phosphatase inhibitors (iNtRON Biotechnology, Korea) and centrifuged at 12,000 × g for 20 min. The total protein concentration was determined using the Bradford assay (Bio-Rad, USA). The samples were resolved in 12% sodium dodecyl sulfate polyacrylamide gel electrophoresis (SDS-PAGE), transferred to 0.45 μm Polyvinyl Difluoride (PVDF) membranes and analyzed separately. After blocking with 5% skim milk at room temperature for 60 min, the blots were surveyed with primary antibodies against rabbit anti-mouse, rat and human fibronectin (1:1000; Santa Cruz, CA), rabbit anti-human α-SMA (1:1000; Abcam), rabbit anti-human, mouse MMP1 (1:1000; Mybiosource), rabbit anti-human, Rat MMP13 (1:1000; Mybiosource), rabbit anti-human p-IκBα (1:1000; Cell Signaling Technology), rabbit anti-human p-ERK (1:1000; Cell Signaling Technology), rabbit anti-human-p-38 (1:1000; Cell Signaling Technology), rabbit anti-human p-JNK (1:1000; Cell Signaling Technology), anti-human p-STAT5 (1:1000; Cell Signaling Technology), anti-human p-IRF3 (1:1000; Cell Signaling Technolog) and rabbit anti-human GAPDH (1:3000; Cell Signal), at 4°C for overnight. Both the NF-κB and MAPKs (which comprise ERK, p38 and JNK) families were activated by IL-25. After serum-starvation for 24 h, the cells were stimulated with IL-25 (100 ng/ml) for 2 h. To evaluate the signaling pathways involved in the induction of α-SMA, fibronectin, MMP-1, and MMP-13 by IL-25 stimulation in the NPDFs, the cells were pretreated for 1 h with U0126 (a specific inhibitor of ERK), SB203580 (a specific inhibitor of p38), SP600125 (a specific inhibitor of JNK inhibitor for JNK1, JNK2 and JNK3), and Bay 11–7082 (NF-κB inhibitor). The cells were then stimulated in the presence of these inhibitors with 100ng/ml of IL-25 for 48 h, and the expression levels of α-SMA, fibronectin, MMP-1, and MMP-13 were determined by western blot assay. The membranes were washed 3 times with TBST buffer (20 mmol/L Tris-buffered saline and 0.1% Tween 20) for 1 h. Peroxidase conjugated anti rabbit-IgG were used as secondary antibodies. Chemiluminescence was performed with the Amersham ECL plus western blotting detection system (GE Healthcare, USA).

### Statistical analysis

The sample size was chosen based on previous studies [3, 20]. We conducted a power analysis that indicated that with 10 patients in both the CRS w NP and the control group, using the standard deviation in the number of double positive cells (Vimentin+ α-SMA+) and a two-sided .05 level of significance, the power was determined to be 0.99.

Statistical analyses were performed using SPSS 22 (version 22.0.0.0, International Business Machines, Armonk, NY, USA) and GraphPad Prism 6 (version 6.01, GraphPad Software, La Jolla, CA, USA). For continuous variables, the results are expressed as mean with standard error of mean (SEM). D’Agostino-Pearson omnibus normality test, Mann-Whitney U test, Kruskal-Wallis tests (2-tailed), and two-way ANOVA were used for the normality test, comparisons of two groups, and multiple testing, respectively. A p value of less than 0.05 was considered statistically significant. The fourth cell passage provided all the cells used for this research. Also, biological and technical triple tests were performed on all of the experiments.

## Results

### Number of myofibroblasts was significantly higher in NP tissues

**Fig 1:**
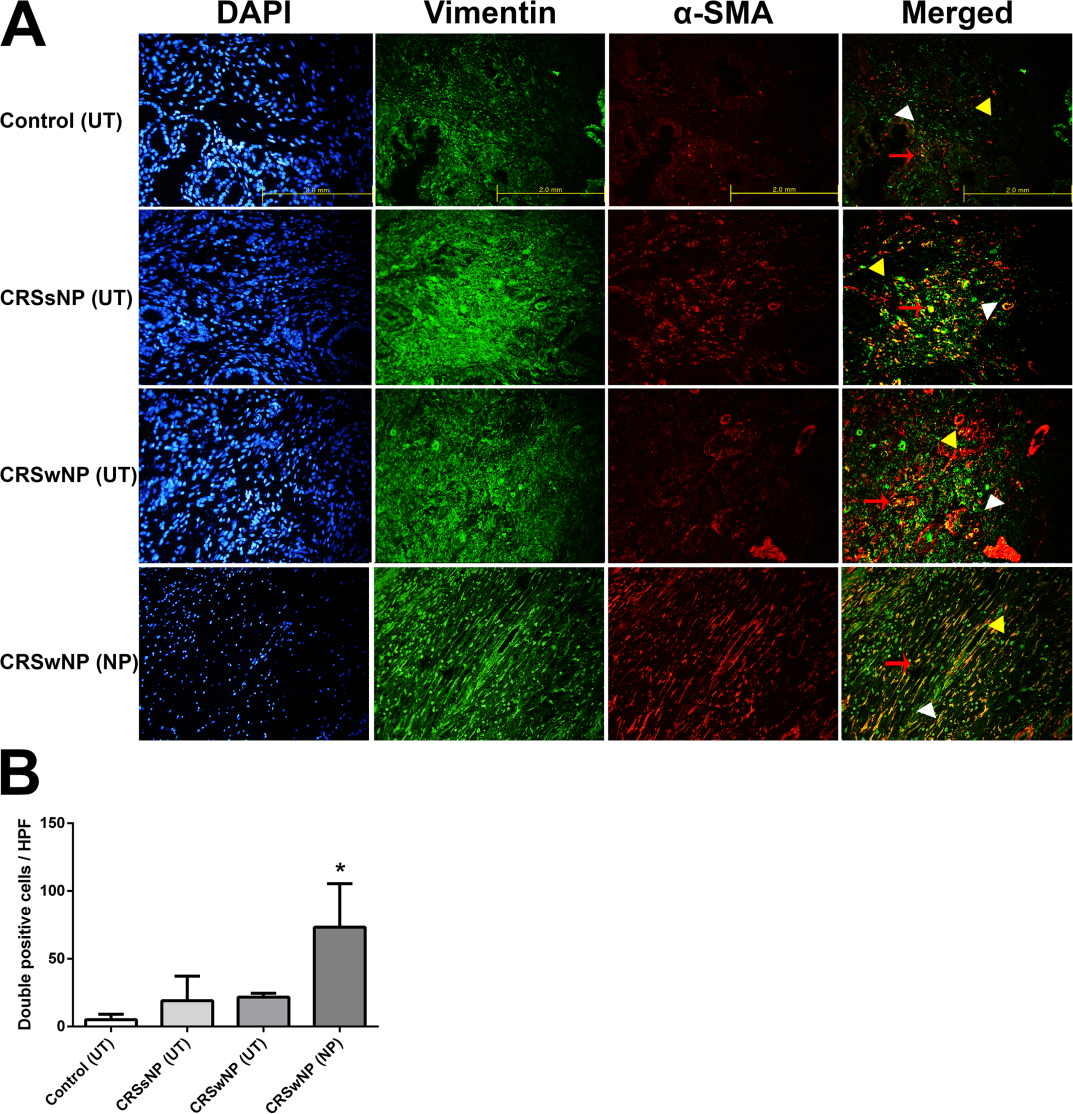
Double–immunofluorescent staining of myofibroblasts in the tissues of the four groups. Double-immunofluorescence staining was undertaken to colocalize the cells with Vimentin (green color)/ α-SMA (red color) among the groups (A). The number of double positive cells (Vimentin+ α-SMA+) was considerably higher in the NP tissues of the CRSwNP group compared to the other groups (*p < 0.05) (B). Vimentin: white arrow head, α-SMA: yellow arrow head, double positive cells: red arrow. Triple tests were performed on all of the experiments.

**Fig 2:**
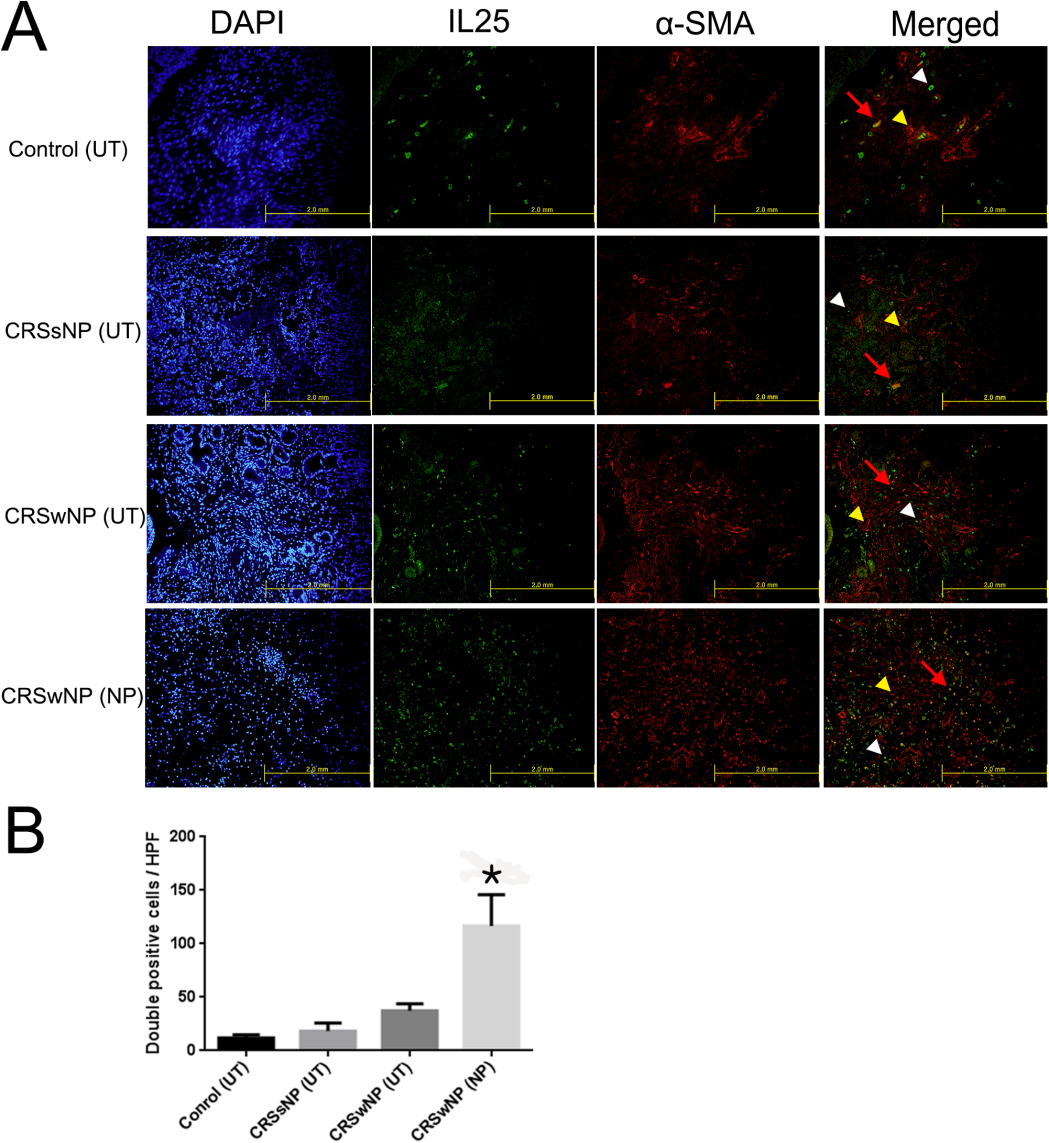
Double–immunofluorescent staining of the correlation between IL-25 level and myofibroblast in the tissues of the four groups. Double-immunofluorescence staining was undertaken to colocalize the cells with IL-25 (green color)/ α-SMA (red color) among the groups (A). The number of double positive cells (IL-25+ α-SMA+) was considerably higher in the NP tissues of CRSwNP group compared to the other groups. Vimentin and IL-25: white arrow head, α-SMA: yellow arrow head, double positive cells: red arrow (*p < 0.05). Triple tests were performed on all of the experiments.

Double immunofluorescent staining (Vimentin/ α-SMA or IL-25/ α-SMA) was conducted to investigate whether myofibroblasts (active form of fibroblast) were involved in pathogenesis of nasal polyp of the CRSwNP group. α-SMA and vimentin expression was observed in cytosol and IL-25 expression was observed in membrane. The number of double positive cells (Vimentin+ α-SMA+ cells) was significantly higher in the NP tissues of the CRSwNP group than in the other groups (p = 0.012) (Fig 1A and 1B). As shown in the above experiment, our FACS analysis using Vimentin (FITC) and α-SMA (APC) divided into four groups confirmed that vimentin was most abundant in the CRSwNP (NP) group (S1 Fig). This result suggests that activation of fibroblasts is associated with nasal polypogenesis in patients with CRSwNP. We then examined the correlation between the IL-25 level and the activated NPDFs in the polyp tissues. Compared to the other group, the number of double positive cells (IL-25+ α-SMA+) in the NP tissues of the CRSwNP group significantly increased (p = 0.016). To show the IL-25 positive cell distribution in the image of the histological sections, we conducted immunohistochemical staining for IL-25, α-SMA and vimentin. Vimentin, α-SMA and IL-25 levels in the patients’ tissues were higher in the CRSwNP (NP) group than in the other 3 groups (S2 Fig). Taken together, these data show that IL-25 induced activation of nasal fibroblast was associated with CRSwNP (Fig 2A and 2B).

### Expression of IL-25 receptor on NPDFs was confirmed by RT-PCR

**Fig 3:**
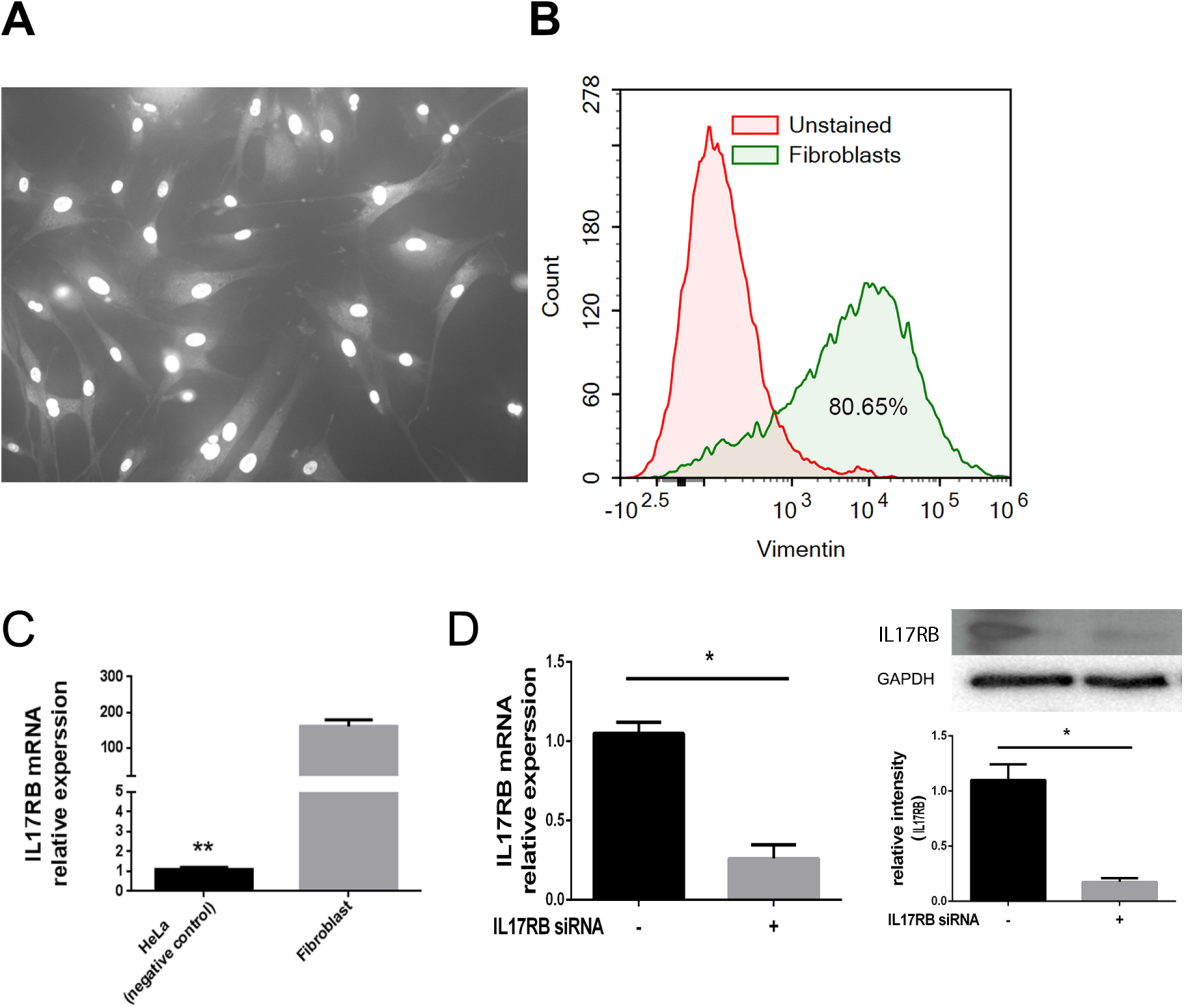
Microscopic morphology of NPDFs and expression of IL-25 receptor on NPDFs. Microscopic morphology of NPDFs (A). Approximately 80% of the cells in the cultured NPDFs were positive for vimentin (B). The mRNA expression of IL-17 receptor B (IL-17RB) was evaluated using RT-PCR (C), the IL-17RB mRNA and IL-17RB protein expression levels were significantly suppressed by IL-17RB siRNA compared with the control siRNA (*p < 0.05) (D). Triple tests were performed on all of the experiments.

Microscopic examination revealed that NPDFs were morphologically bipolar or multipolar, with elongated shapes, and were growing attached to a substrate (Fig 3A). Approximately 80% of the cells in the cultured NPDFs tested positive for vimentin, which was used as a fibroblast marker (Fig 3B). To determine whether the IL-25 receptor was involved in the IL-25-induced ECM and MMP expressions in the NPDFs, mRNA expression of IL-17 receptor B (IL-17RB) was evaluated using RT-PCR. IL-17RB mRNA levels were also measured in HeLa cells (negative control), CRSwNP(NP) and control (UT). We found that IL-17RB mRNA was expressed on the NPDFs. In addition, IL-17RB mRNA was expressed more in NPDFs than in HeLa cells, and it was found to be less than in CRSwNP (NP) and control (UT) tissues. (Fig 3C). We measured IL-17RB mRNA and IL-17RB protein expression levels with IL-17RB siRNA (p < 0.001) and confirmed that IL-17RB mRNA and IL-17RB protein expression was suppressed by approximately 60% and 80%, respectively, by IL-17RB siRNA (Fig 3D).

### IL-25 induced myofibroblast differentiation (α-SMA), fibronectin production and MMP-1 and -13 expressions in NPDFs

**Fig 4:**
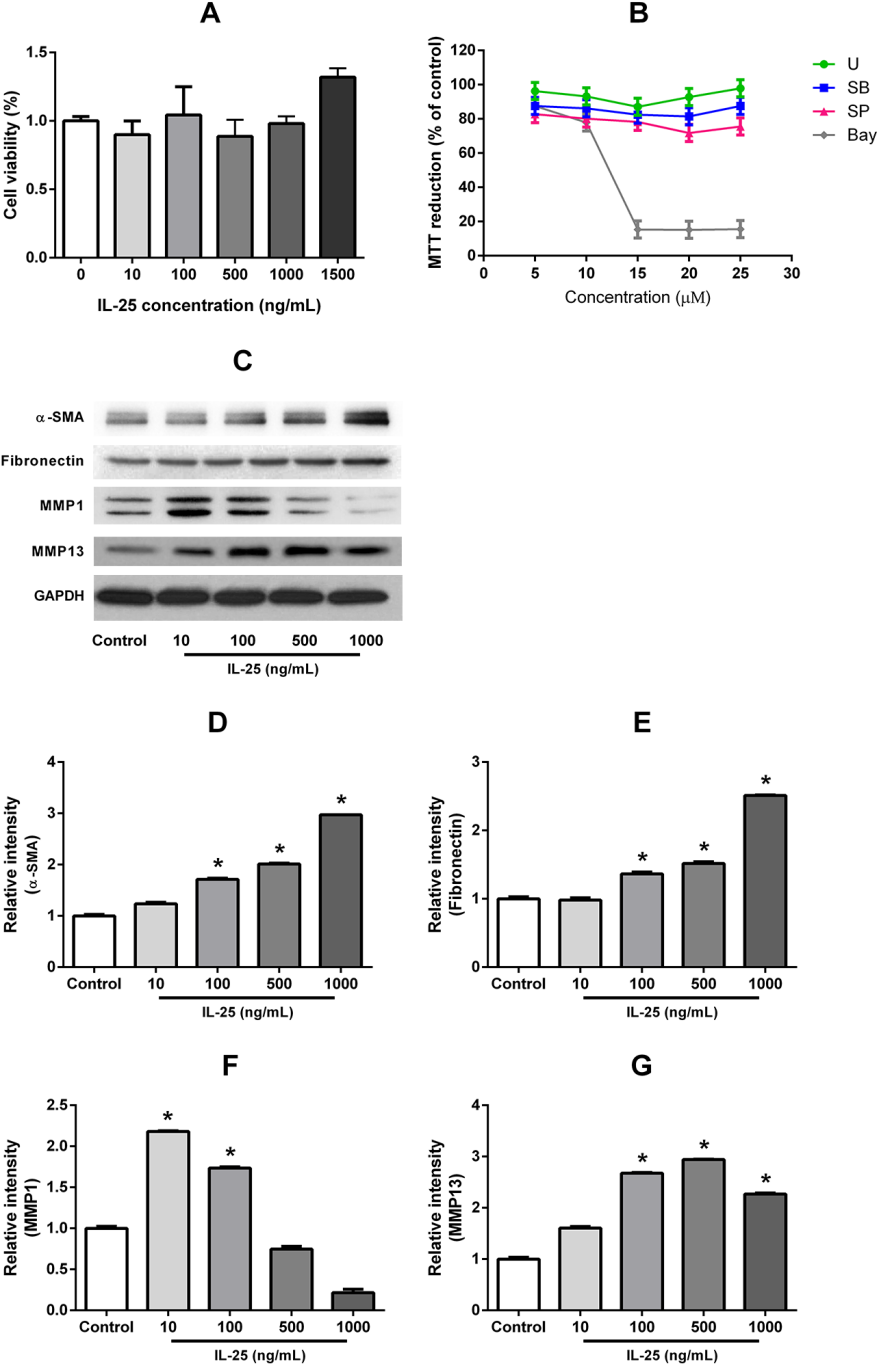
IL-25 induced myofibroblast differentiation (α-SMA), fibronectin production and MMPs expression in NPDFs. An MTT assay was performed to exclude the possibility of IL-25-induced and specific inhibitors-induced cellular cytotoxicity of the NPDFs (A). The specific inhibitors did not affect cell viability (B) The expression levels of α-SMA, fibronectin, MMP-1 and MMP-13 protein were determined by western blot assays (*p < 0.05) (C-G). Triple tests were performed on all of the experiments.

We found that IL-25 did not directly influence the survival of the NPDFs at doses ranging between 10 and 1500 ng/ml for 72 hours (Fig 4A). U (specific inhibitor of ERK), SB(specific inhibitor of p38), SP (specific inhibitor of JNK) and Bay(specific inhibitor of NF-κB) also did not affect cell viability until the concentration reached 10μg/mL for 72hours (Fig 4B). The expression levels of α-SMA, fibronectin and MMP-13 protein were determined by western blot assays which revealed that the expression of these proteins was significantly increased after treatment with various concentrations (10-1500ng/mL) of IL-25 (p < 0.0001) (Fig 4C, 4D, 4E and 4G). The expression of MMP-1 was increased at 10 and 100 ng/ml of IL-25 stimulation, but reduced at 1000 and 1500 ng/ml of IL-25 stimulation (p < 0.0001) (Fig 4C and 4F). These results indicate a dose dependent manner in which IL-25 induced myofibroblast differentiation (α-SMA) ECM production, and MMP-13 expression occur.

### IL-25 induced activation of MAPKs and NF-κB in NPDFs

**Fig 5:**
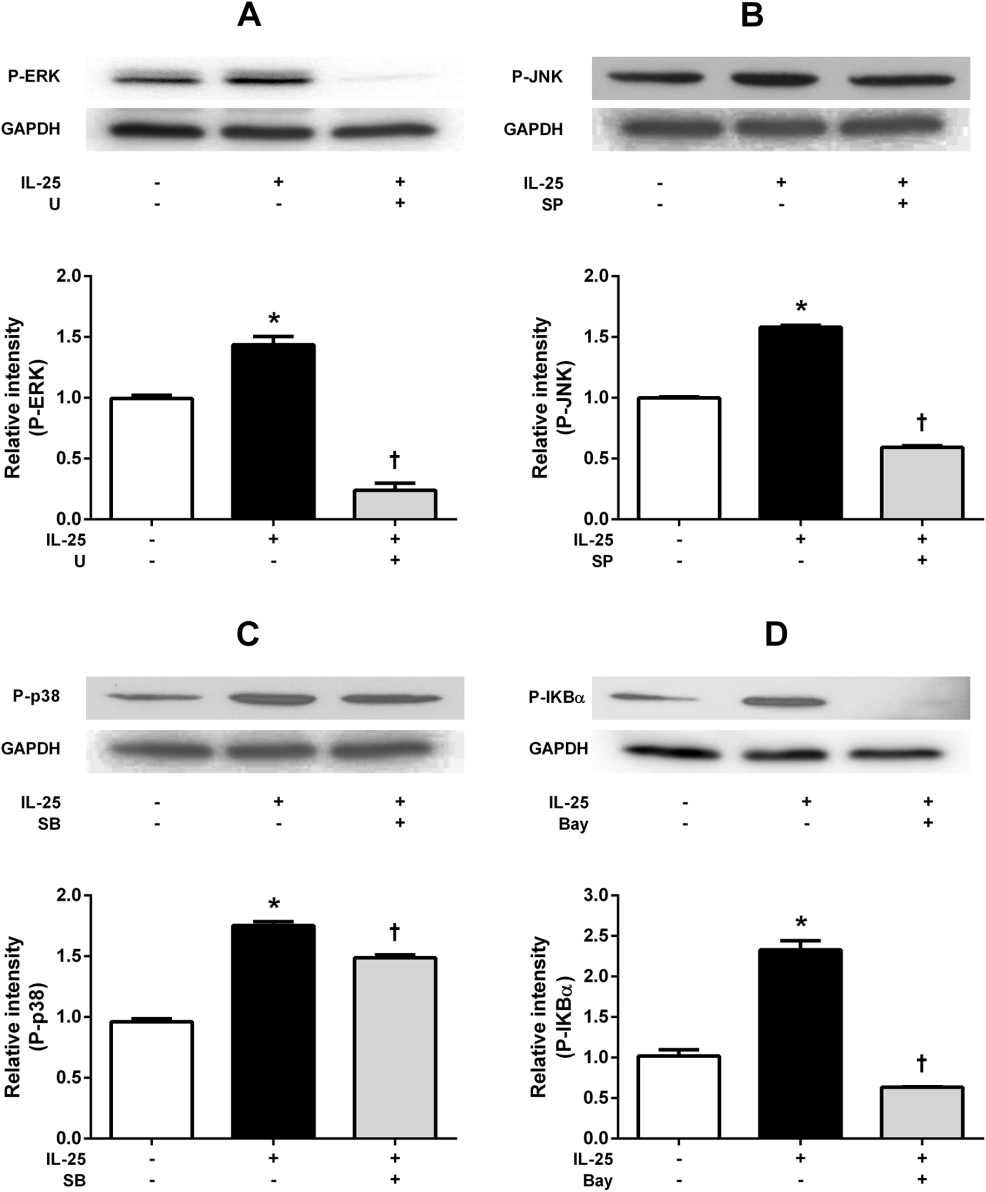
IL-25 induced activation of MAPKs and NF-κB in NPDF. Effects of IL-25 on the activation of mitogen-activated protein kinases (ERK, p38, JNK) and NF-κB evaluated by Western blotting. Phosphorylation of representative MAPKs, ERK (A), JNK (B), and p38 MAPK (C) was induced by IL-25 treatment, after which they became inhibited by their specific inhibitors, including U, SP, and SB, respectively. (p < 0.05). IL-25 induced phosphorylation of IκBα; p-IκBα expression was inhibited by Bay (p < 0.05) (D). Values are expressed as means ± standard errors of independent experiments. (*p < 0.05 vs control). †p <0.05 vs IL-25 alone. Triple tests were performed on all of the experiments. U = specific inhibitor of ERK, SB = specific inhibitor of p38, SP = specific inhibitor of JNK, Bay = specific inhibitor of U = specific inhibitor of NF-κB.

Activations of NF-κB and MAPKs has been shown to play essential roles in IL-25R-mediated cellular activation and gene expression in a number of different cell types [19, 21–23]. As representative MAPKs, phosphorylation of ERK, JNK, and p38 MAPK were also induced by IL-25 treatment, and the use of U (specific inhibitor of ERK), SB (specific inhibitor of p38) and SP (specific inhibitor of JNK) showed a markedly reduced expression of p-ERK, p-p38, and p-JNK. (p-p38 reduced expression p value is 0.0028; the others are p < 0.001) (Fig 5A–5C). IL-25 also induced phosphorylation of IκBα, a frequently used marker for the activation of the NF-κB pathway, which significantly decreased when pretreated with Bay (p < 0.0001) (Fig 5D). Taken together, our data indicates that IL-25 induces activation of NF- κB and MAPKs in NPDFs.

### IL-25-induced α-SMA, fibronectin and MMPs expressions are differentially regulated through various signaling pathway in NPDFs

**Fig 6:**
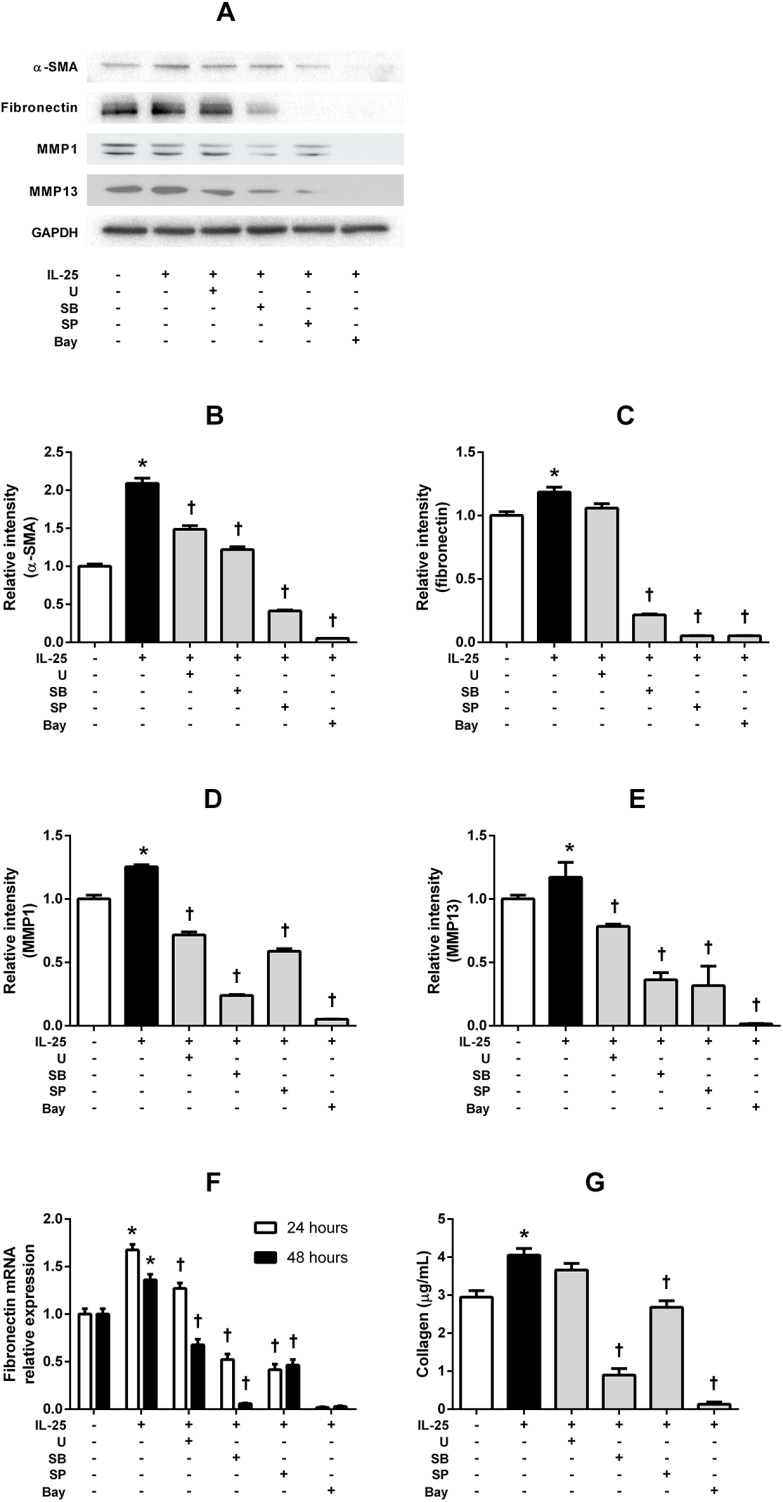
**Regulation of IL-25-induced α-SMA, fibronectin and MMPs expression through various signaling pathways in NPDFs.** (A) The expression levels of α-SMA, fibronectin, MMP-1, and MMP-13 were determined by western blot assay or RT-PCR. Treatments with the specific inhibitors significantly inhibited IL-25-induced α-SMA (B), MMP-1 (D), and MMP-13 (E) protein expression (p < 0.05). IL-25-induced fibronectin (C) expression was inhibited by pretreatment with NF-κB, JNK, and p38 pathway inhibitors, but not by the ERK inhibitor. IL-25-induced fibronectin mRNA expression level was measured by RT- PCR (F). The total soluble collagen level was measured by collagen assay (G) Values are expressed as means ± standard errors of independent experiments. *P<0.05 vs control; †p <0.05 vs IL-25 alone. Triple tests were performed on all of the experiments. U = specific inhibitor of ERK, SB = specific inhibitor of p38, SP = specific inhibitor of JNK, Bay = specific inhibitor of U = specific inhibitor of NF-κB.

Treatment with the specific inhibitors significantly inhibited IL-25-induced α-SMA, MMP-1, and MMP-13 protein expressions. Treatment with the significantly inhibited IL-25-induced α-SMA protein expressions (p < 0.0001), (Fig 6A, 6B, 6D and 6E). IL-25-induced fibronectin expression was inhibited by pretreatment with the NF-κB, JNK, and p38 pathway inhibitors (p < 0.0001), but not by the ERK inhibitor (p = 0.02) (Fig 6A and 6C). However, with pretreatment of the ERK inhibitor, the IL-25-induced fibronectin mRNA expression was significantly inhibited (p < 0.0001) (Fig 6F). Similar results were observed for soluble total collagen levels, and collagen production induced by IL-25 was inhibited by all the inhibitors (p < 0.0001) (Fig 6G). These results indicate that IL-25-induced myofibroblast differentiation, ECM production and MMP expression are related to the signaling pathways of MAPKs, and NF-κB, although to differing degrees. We can further report that NF-κB is the main signaling pathway in IL-25-induced myofibroblast differentiation, ECM production and MMP expression.

### IL-25 induced α-SMA, fibronectin and MMPs expressions through IL-17RB-dependent pathways in NPDFs

**Fig 7:**
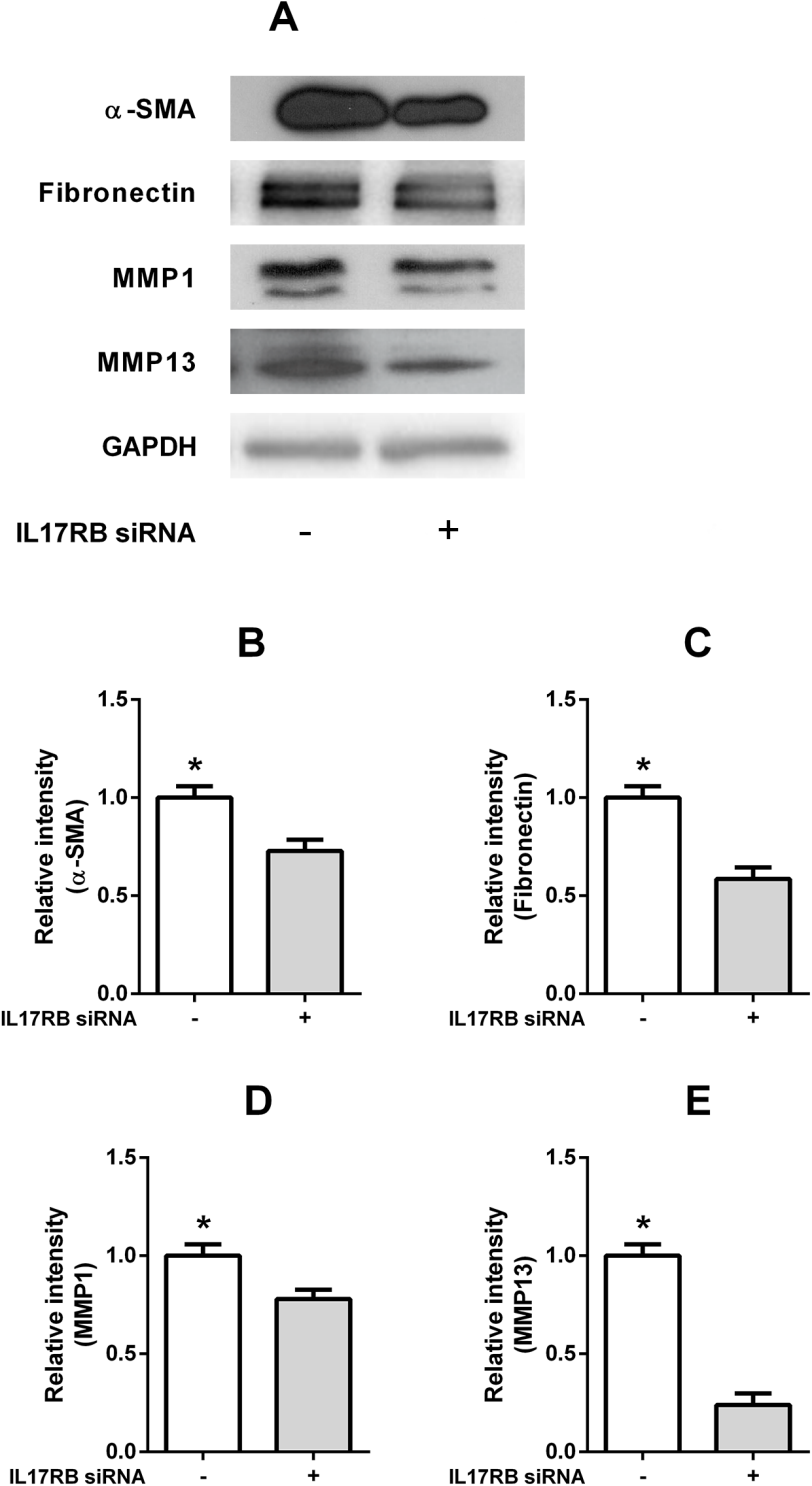
IL-25 induced α-SMA, fibronectin and MMPs expressions via IL-17RB dependent pathways in NPDFs. Knock down of IL-17RB by IL-17RB siRNA decreased IL-25-induced a-SMA, fibronectin and MMPs expression in NPDFs (A, B, C, D, E). Values are expressed as means ± standard errors of independent experiments. *P<0.05 vs control. Triple tests were performed on all of the experiments.

To assess whether IL-25-induced α-SMA, fibronectin and MMPs expressions depend on the IL-25 receptor signal pathway, we suppressed IL-25R expression by IL-17RB siRNA (Fig 3D). Knock down of IL-17RB decreased IL-25-induced expression of α-SMA, fibronectin and MMP in the NPDFs (p = 0.0292, 0.0071, 0.00421 and 0.0007 respectively), and the expression levels were determined by western blot assay (Fig 7A–7E). These data indicate that IL-25 the expression of induces α-SMA, fibronectin, and MMPs proteins through IL-17RB-dependent pathways in NPDFs, and the expression levels were determined by western blot assay.

### IL-25 facilitated migration of NPDFs

**Fig 8:**
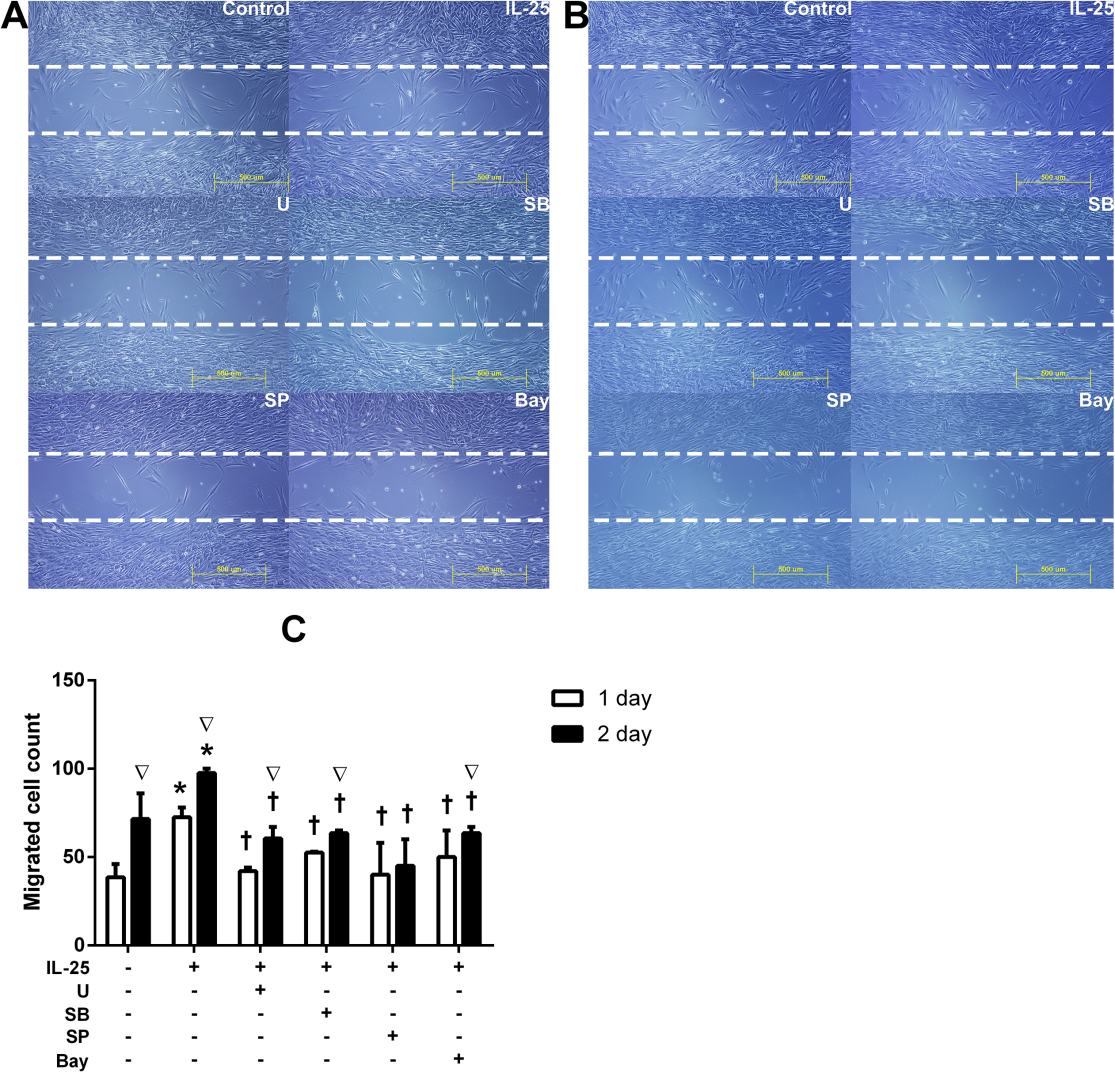
Migration of NPDFs facilitated by IL-25 facilitated the migration of NPDFs. Migration cells were examined by a cell migration assay. Photos of our microscopic observations of cell migration assays showing nasal fibroblast migration by IL-25 and pretreatment with specific inhibitors after 24h (A) and 48h (BC). The number of fibroblasts was counted in a ‘cell-free gap’ (500μm in width) after 24h and 48h (C). Values are expressed as means ± standard errors of independent experiments. *P<0.05 vs control; †p <0.05 vs IL-25 alone; ∇p <0.05 vs 24hr. Scale bar = 500 μm; Triple tests were performed on all of the experiments. U = specific inhibitor of ERK, SB = specific inhibitor of p38, SP = specific inhibitor of JNK, Bay = specific inhibitor of U = specific inhibitor of NF-κB.

To examine the IL-25-induced influence of migration in NPDFs, cell migration assays were performed. The number of fibroblasts was counted in a “cell-free gap” (500μm in width) after 24 and 48hours. Compared to the controls, the number of fibroblasts that had migrated into the cell-free gap was significantly higher in the cells treated with IL-25. Pretreatment with the specific inhibitors for signaling molecules significantly decreased the number of migrated cells (p < 0.05) (Fig 8).

## Discussion

Chronic rhinosinusitis (CRS) is associated with severe inflammation, and CRSwNP may overwhelmingly lead to severe edematous change, while CRSsNP usually exhibits fibrotic remodeling of the lamina propria [24]. Although CRSwNP and CRSsNP present predominant edematous and fibrotic patterns, respectively, a recent study demonstrated that many aspects of the changes in tissue composition are not specific to the diseases and can present a wide range of severity [25]. The precise molecular factors mediating this differential remodeling pattern are not fully understood, but a recent study has suggested that TGF-β1 may play a key role in this process; this study reported that low levels of TGF-β1 were observed in CRSwNP while high levels were observed in CRSsNP [26]. Another study, however, reported higher expressions of TGF-β1 in the nasal secretions of CRSwNP, than in the control or CRSsNP patients [3, 27].

Despite differing reports regarding the expression levels of TGF-β1 or patterns of connective tissue composition in CRSwNP, it is generally understood that the expression of IL-25 is significantly higher in the nasal polyps of CRSwNP patients than in CRSsNP patients or control subjects [3]. As IL-25 is generally considered to be the dominant innate cytokine with a critical role in the pathogenesis of polyp formation in CRSwNP, [13] and because expression of IL-25 is known to be involved in airway remodeling, [19] in the present study, we examined whether IL-25 influences tissue remodeling in CRSwNP. We also evaluated the effects of IL-25 on the activations of fibroblasts, one of the main components of nasal polyps and a main determiner of tissue composition. We demonstrated that IL-25 induced an increased production of collagen and fibronectin as well as expression of MMPs through the NF-κB and MAPKs signaling pathway in the NPDFs.

IL-25, also known as IL-17E, is mainly produced by mucosal epithelial cells. Over-expression of IL-25 is associated with increasing eosinophilia and a TH2 dominant immune response. Although it has been demonstrated that IL-25 receptor (IL-25R) is composed of IL-17RB and IL-17RA and forms a functional complex, the specific contribution of each subunit to downstream signaling remains unclear [16]. Ongoing studies have indicated that IL-17RB is expressed by various cell types such as epithelial cells, T cells, monocytes and innate lymphoid cells, [28–30] but studies of IL-25 have been focused only on their immunologic functions on T cells or various inflammatory granulocytes, and the expression of IL-17RB on fibroblasts is yet to be investigated. We have demonstrated that IL-17BR is also expressed on NPDFs and induces activation of the fibroblasts, as well as ECMs and MMPs production. To our knowledge, our study is the first to report that IL-25 activates nasal fibroblasts and thereby induces myofibroblast differentiation and ECMs and MMPs production in nasal polyps.

IL-17RB encodes a TRAF6-binding motif in its cytoplasmic tail. Antibody-mediated crosslinking of the receptor activates NF-κB, which can be blocked by a dominant-negative form of TRAF6 [15, 22]. The IL-17RB cytoplasmic tail contains a SEFIR domain, and was recently shown to bind ACT1 in a SEFIR-dependent manner [15]. Apart from signaling through Act1 and TRAF6, IL-25 has been shown to activate MAPKs such as p38 and JNK as well as a NF-κB in eosinophils [22].

The MAPK pathways consist of many phosphorylation cascades, each of which modulates different signaling event, either alone or in combination. The MAPK pathways have been reported as Smad-independent TGF-β signaling pathways [8, 31], ERK, JNK, and p38 are known to participate in TGF-β signaling cascades [8, 32]. MAPK activation regulates phosphorylation of the down-stream transcription factor NF-κB, which leads to the activation of the NF-κB signaling pathway [8, 33]. We used the inhibitors U0126 (a specific inhibitor of ERK, 10μM), SB203580 (a specific inhibitor of p38, 10μM), SP600125 (a specific inhibitor of JNK, 10μM), and Bay 11–7082 (NF-κB inhibitor, 1μM) to elucidate the intracellular signaling mechanisms regulating the induction of the fibroblast activation. Based on previous publications and toxicity results we used the optimal concentrations of U0126 (a specific inhibitor of ERK, 10μM), SB203580 (a specific inhibitor of p38, 10μM), SP600125 (a specific inhibitor of JNK, 10μM), and Bay 11–7082 (NF-κB inhibitor, 1μM) with the highest inhibitory effect without any cell toxicity [8]. Our inhibition experiments have demonstrated that IL-25-induced fibroblast activation and ECM and MMPs production were mediated by NF-κB, p38, and JNK activities. In other words, IL-25 induced the migration ability of the fibroblast, which was mediated by the NF-κB, p38, and JNK signal pathways. In addition, the NF-κB pathway was the transcription factor most involved in the IL-25-induced activation of the NPDFs. In our study, the production of fibronectin and collagen was not significantly inhibited by the ERK inhibitor, which indicates that ERK is not involved in the production of fibronectin and collagen. However, the production of α-SMA, MMP-1, and -13, and migration ability was associated with the ERK signaling pathway.

Current studies in this field have demonstrated that MMPs and TIMPs play an essential role in tissue remodeling, [34–36] as described in detail previously [34], this is because increased expression of MMPs or reduced TIMP-1 could induce ECM rupture and promote deposits of substances such as water and albumin. This ECM remodeling could result in edema and trigger a local inflammatory process, [37]. Therefore, it would be reasonable to hypothesize that MMPs expression could be a factor in a more pronounced edema and consequently larger disease extension. Indeed MMPs and TIMP expression levels iffer between CRSwNP and CRSsNP patients, suggesting that MMPs could be pivotal in worsening wound healing and promoting tissue edema in nasal polyps [38]. Myofibroblasts are known to play a crucial role in MMPs production in the tissues, and evidence to support the hypothesis that fibroblasts perform a crucial role by differentiating into myofibroblasts has been reported [39]. Myofibroblasts, which express α-SMA, produce a large quantity of ECM components. This process results in the accumulation of ECM and has an important impact on the structural modification of nasal polyps [40]. Our results indicate that IL-25, one of the dominant cytokines in Asian nasal polyps, also induce the expression of α-SMA in NPDFs in a dose-dependent manner.

It is widely known that various substance, such as cell adhesive molecules and ECM-like type IV collagen, type VII collagen, laminin, fibronectin, and heparin sulfate comprise the mucosal basement membrane, which could be destroyed by various MMPs [41, 42]. In addition, a thickening of the layer below the basal lamina consisting of collagens type I, III, and V plus fibronectin is a hallmark of remodeling in asthma [43]. At least 23 MMP family members have been characterized [44, 45]. In particular, MMP-13 is thought to play key roles in tissue remodeling and repair through the degradation of type IV collagen, a major component of the basement membrane zone [46]. Malinsky et al. reported a higher expression of MMP-1 mRNA in patients with nasal polyps than in the controls, suggesting that MMP-1 (also known as collagenase 1 and known to initiate degradation of type I and III fibrillar collagen) could play an important role in CRSwNP [34]. MMP-13 (precursor of collagenase 3) degrades fibrillar collagen found in extensible connective tissues such as the skin, lungs, and vascular system, frequently in association with type I collagen [47, 48]. Molet et al. demonstrated that collagen types I, III, and V were increased in all nasal polyp tissues, with a predominance of types III and V [49]. Muro et al. [50] and Huang et al. [51] reported that in diseases such as allergic rhinitis and sinusitis, histological evidence of ECM remodeling includes basement membrane thickening, subepithelial fibrosis, with increased deposition of collagen type I and III along with other matrix products. Therefore, we evaluated IL-25-induced expressions of MMP-1 and MMP-13 on NPDFs and found that IL-25 increased the MMP-1 and MMP-13 expressions and that this process was mediated by intracellular MAPKs and NF-kB activation. De Borja et al. [52, 53] reported the expression of MMPs and TIMPs in different tissue structures and cells from NP and nasal mucosa, and included a variety of MMPs and TIMP in their experiments. The present study has several limitations. We did not conduct any studies on TIMPs that regulate MMPs, and only a few types of MMPs related to nasal polyps are evaluated in this study. In addition, other cytokines secreted in association with IL-25 are not included. Inclusion of other cytokines would help to clarify the IL-25 associated signaling.

## Conclusions

This is the first study on the activation of NPDFs for the release of ECM proteins and MMPs by IL-25. Our results suggest that IL-25-induced release of α-SMA, fibronectin, collagen, MMP-1 and MMP-13 from NPDFs is mediated by the combined activation of the MAPK and NF-kB pathways, thereby providing new clues for fibroblast-mediated inflammation by changing the ECM composition in nasal polyps. Further investigations are required for other potential intracellular signaling pathways for the regulation of the release of ECM and MMPs.

## Supporting information

S1 Fig A flow cytometric analysis of double positive cells between Vimentin and α-SMA. (TIF)

S2 Fig Immunohistochemistry staining pictures for IL25, a-SMA and vimentin. (TIF)
