# Supplementary figures and images for: IL-25-induced activation of nasal fibroblast and its association with the remodeling of chronic rhinosinusitis with nasal polyposis
Source: PLoS One. 2017 Aug 3;12(8):e0181806. doi: 10.1371/journal.pone.0181806 (PMC5542454; doi:10.1371/journal.pone.0181806)

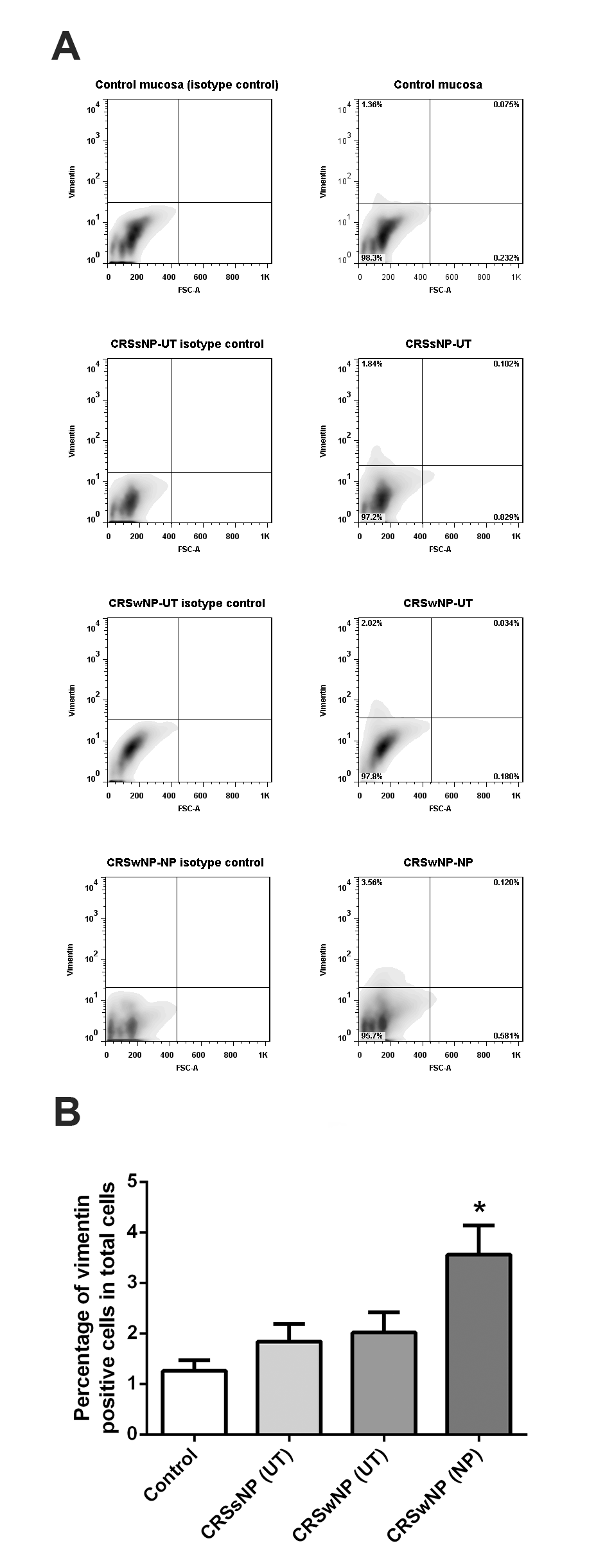

Supplement: S1 Fig — (TIF) [file pone.0181806.s001.tif]

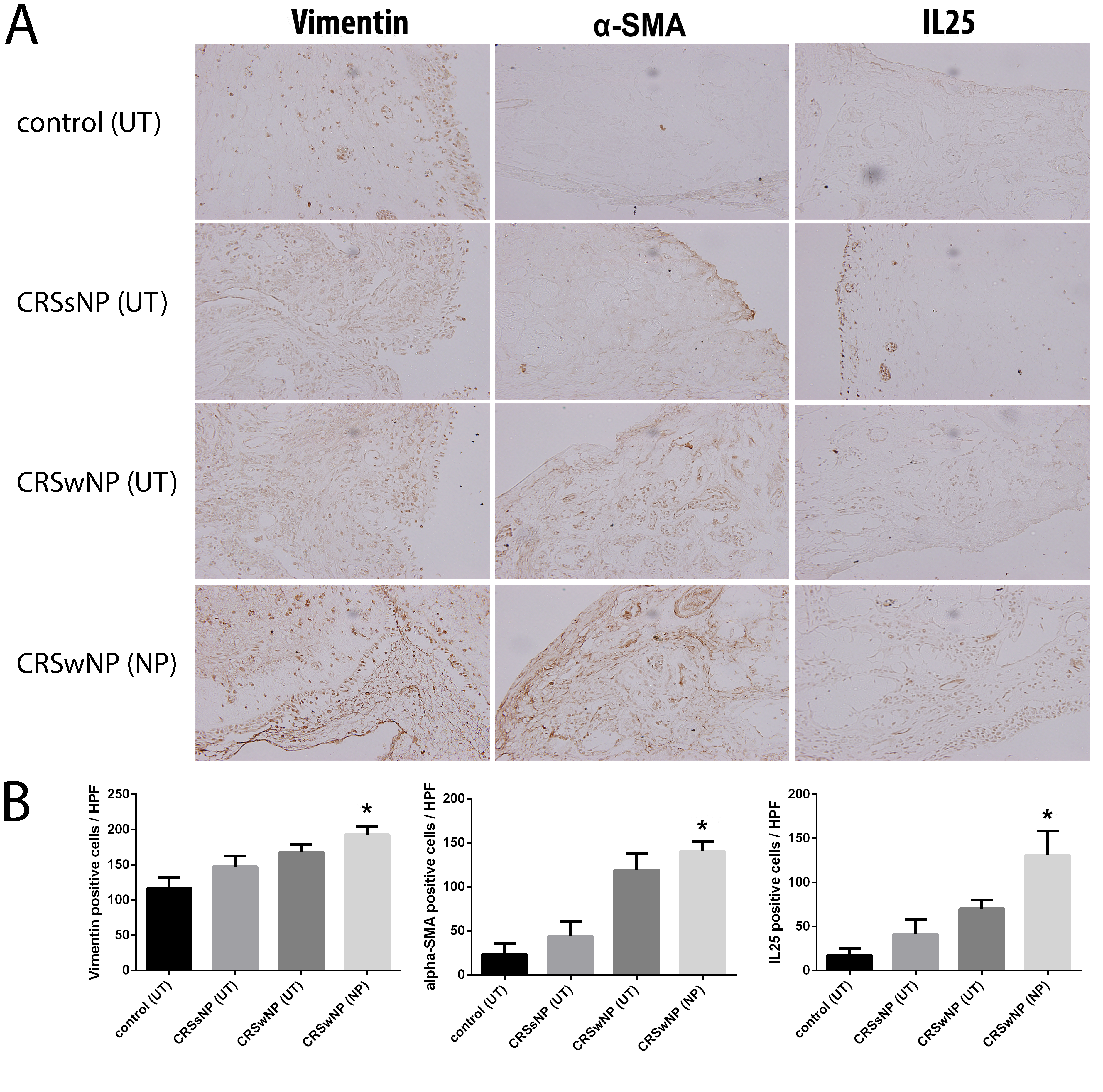

Supplement: S2 Fig — (TIF) [file pone.0181806.s002.tif]
